# Nonlinear changes in urban heat island intensity, urban breeze intensity, and urban air pollutant concentration with roof albedo

**DOI:** 10.1038/s41598-024-76935-4

**Published:** 2024-10-22

**Authors:** Kyeongjoo Park, Jong-Jin Baik

**Affiliations:** School of Earth and Environmental Sciences, Seoul National University, 08826 Seoul, South Korea

**Keywords:** Roof albedo, Urban heat island, Urban breeze circulation, Pollutant dispersion, Urban air quality, Cool roof, Environmental impact, Atmospheric science

## Abstract

This study systematically examines how the urban heat island (UHI) and urban breeze circulation (UBC) respond to an increase in roof albedo (*α*_r_) and its influence on urban air pollutant dispersion. For this, idealized ensemble simulations are performed using the Weather Research and Forecasting (WRF) model. The increase in *α*_r_ from 0.20 to 0.65 decreases the UHI intensity, UBC intensity, and urban planetary boundary layer (PBL) height in the daytime (from 1200 to 1700 LST) by 47%, 36%, and 6%, respectively. As both UBC intensity and urban PBL height decrease, the daytime urban near-surface passive tracer concentration increases by 115%. The daytime UHI intensity, UBC intensity, and urban tracer concentration nonlinearly change with *α*_r_: For 0.10 ≤ *α*_r_ < 0.80, the rates of changes in the UHI intensity, UBC intensity, and urban tracer concentration with *α*_r_ overall increase as *α*_r_ increases. For *α*_r_ ≥ 0.80, the daytime roof surface temperature is notably lower than the daytime urban near-surface air temperature, the UHI intensity, UBC intensity, and urban tracer concentration very slightly changing with *α*_r_. This study provides insights into the associations between changes in roof surface temperature and roof surface energy fluxes with *α*_r_ and those in UHI intensity.

## Introduction

Although urban areas account for only about 3% of the world’s total land area, urban areas now house more than 50% of the world’s population^1,2^. Dense populations and numerous infrastructures in urban areas result in significant modifications in local climates^3,4^. In particular, urban areas typically exhibit higher near-surface air temperature than their surrounding rural areas, which is called the urban heat island (UHI)^5^. The UHI is known to have adverse health effects on urban residents in warm seasons^6,7^. According to Lowe^8^, the UHI may increase heat-related deaths by 1.1 people per million in U.S. cities. Moreover, the adverse effects of the UHI are expected to be intensified with climate change and increasing heat wave occurrences, which necessitates an effective UHI mitigation strategy^9,10^.

To alleviate the UHI, various mitigation strategies have been suggested^11^. Among them, the cool roof (reflective roof) is one of the most widely adopted UHI mitigation measures^12^. The cool roof is referred to as a roof with higher albedo and emissivity than conventional roofs^13^. Many previous studies showed that the application of cool roofs effectively decreases the UHI intensity^14–22^. For instance, He et al.^15^ examined cool-roof effects on the UHI in 16 cities in the Yangtze River Delta, China and showed that increasing roof albedo from 0.12 to 0.65 decreases urban 2-m temperature by up to 1.19 °C in summer. Reed and Sun^20^ investigated cool-roof scenarios in Kansas City, U.S. and revealed that increasing roof albedo from 0.3 to 0.8 decreases the UHI intensity by up to 0.64 °C. Baik et al.^14^ examined cool-roof effects on urban thermal and wind environments during a record-breaking heat wave event in Seoul, South Korea and showed that increasing roof albedo from 0.2 to 0.7 decreases urban 2-m temperature by up to 1.0 °C. Zonato et al.^23^ examined how cool-roof effects on the UHI differ depending on urban morphology and season through idealized simulations. They revealed that cool-roof effects on the UHI are enhanced with increasing the plan area of buildings and with decreasing building height and are stronger by about 10 times in summer than in winter.

Meanwhile, some previous studies showed that the application of cool roofs may reduce ventilation and therefore worsen air quality in urban areas^24–28^. Wang et al.^26^ examined cool-roof effects on the concentration of particulate matter with a diameter less than 2.5 μm (PM_2.5_) in North China and revealed that increasing roof albedo from 0.2 to 0.9 increases urban PM_2.5_ concentration in the daytime by up to 42%. Zhong et al.^28^ evaluated cool-roof effects on heavy ozone (O_3_) pollution events in Shanghai, China and showed that the application of cool roofs increases urban near-surface O_3_ concentration by suppressing the development of planetary boundary layer (PBL). On the other hand, it was also reported that the application of cool roofs may decrease urban O_3_ concentration by reducing the rates of temperature-dependent reactions and energy consumption which causes the emission of O_3_ precursors^29,30^.

The alleviation of the UHI is a beneficial effect of cool roofs in terms of thermal discomfort in warm seasons, but the air stagnation and thus the deterioration of air quality are side effects that could be accompanied by the application of cool roofs^31^. In order to find an appropriate trade-off between the UHI mitigation and its side effects on air quality, how the UHI responds to an increase in roof albedo and how this affects urban air quality should be well understood but are still open questions. Several case studies reported an almost linear relationship between the UHI intensity and roof albedo^27,32,33^. For instance, Li et al.^32^ simulated a heat wave event in Baltimore, U.S. using the Weather Research and Forecasting (WRF) model and revealed that the UHI intensity almost linearly decreases with increasing roof albedo. On the other hand, a few case studies revealed a nonlinear relationship between the UHI intensity and roof albedo^16,17^. Imran et al.^16^ examined three cool-roof scenarios with roof albedos of 0.50, 0.70, and 0.85 during a heat wave event in Melbourne, Australia and showed that the increase in roof albedo from 0.50 to 0.70 has a larger UHI mitigation effect than that from 0.70 to 0.85.

Despite many attempts to understand the relationships of the UHI and urban air quality with roof albedo in various cities, most previous studies have examined only a few roof albedo cases or a limited range of roof albedo. Furthermore, the urban breeze circulation (UBC), which is a mesoscale circulation induced by the UHI^34^, is expected to be significantly modified as the roof albedo changes^28^, but the relationship between the UBC and roof albedo has been rarely examined. Given its significant influences on local circulations and air quality in urban areas^35,36^, a detailed examination of changes in the UBC with roof albedo could enhance the understanding of roof albedo effects on urban environments. Thus, in this study, we aim to systematically examine how the UHI and UBC respond to an increase in roof albedo and how this affects urban air pollutant dispersion. For this, idealized ensemble simulations with various roof albedos from 0.10 to 0.95 are performed. The idealized simulations enable to examine various roof albedo cases because of their low computational cost^23^. Furthermore, in the idealized simulations, the interferences of synoptic and local features can be excluded^37^, making it possible to find essentially important physical processes for changes in the UHI, UBC, and urban air pollutant dispersion with roof albedo.

## Methods

This study uses the WRF model version 4.1.3^38^ to perform the idealized ensemble simulations. This study considers an idealized two-dimensional (*x*–*z*) computational domain with the horizontal and vertical sizes of 500 km and 7 km, respectively. The 250-m horizontal grid interval is employed, and the vertical grid interval increases with height. The height of the first model level is 13 m, and the number of vertical layers is 66. The periodic lateral boundary conditions are adopted, and the Rayleigh damping^39^ is applied above 5 km. An urban area is considered in the middle area of the domain, and its size is 20 km. The land use types of the urban area and surrounding rural area are industrial/commercial area and cropland/woodland mosaic, respectively. The midlatitude (30°N) is considered.

To better simulate urban land surface processes, the Seoul National University Urban Canopy Model (SNUUCM)^40^ is coupled with the WRF model. The SNUUCM is one of the single-layer urban canopy models (UCMs) that can be implemented in atmospheric models. The key features of the SNUUCM are that two building walls (sunlit and shaded walls) are separately treated and that the effects of reference wind direction and canyon aspect ratio are considered in calculating the canyon wind speed^40^. The SNUUCM well simulates surface energy fluxes and the surface temperatures of urban facets observed in many urban sites^40–43^. In the urban grid, the fraction of built-up area is set to 0.9, and the fraction of natural area is set to 0.1. To simulate land surface processes in the rural grid and the natural area fraction in the urban grid, the unified Noah land surface model^44^ is used. Both road width and building height are set to 10 m, the urban canyon aspect ratio being 1.0^45^. The roof width is set to 10 m as well. The emissivity, heat capacity, thermal conductivity, and roughness length of roof are set to 0.95, 1.0 MJ m^−3^ K^−1^, 0.67 J m^−1^ s^−1^ K^−1^, and 0.01 m, respectively. The albedos/emissivities of both road and walls are 0.18/0.95, and the heat capacity, thermal conductivity, and roughness length of road (walls) are 1.4 MJ m^−3^ K^−1^ (1.0 MJ m^−3^ K^−1^), 0.40 J m^−1^ s^−1^ K^−1^ (0.67 J m^−1^ s^−1^ K^−1^), and 0.01 m (0.0001 m), respectively^45^. For PBL and surface layer parameterizations, the Yonsei University PBL scheme^46^ and the revised MM5 similarity scheme^47^ are employed, respectively. For cloud microphysics parameterization, the WRF single-moment 6-class scheme^48^ is used. Note that cloud microphysical processes do not affect our main results since the clear-sky (cloudless) conditions are considered in this study. For radiation parameterizations, the Dudhia shortwave radiation scheme^49^ and the Rapid Radiative Transfer Model (RRTM) longwave radiation scheme^50^ are used. To investigate the impact of increasing roof albedo on urban air pollutant dispersion, the WRF model is coupled with chemistry^51^, a passive tracer option being used. In this option, the carbon monoxide (CO) is treated as a passive tracer and its transport is simulated.

Total 18 experiments are conducted by applying different roof albedos from 0.10 to 0.95 with an interval of 0.05. For initial sounding, a linear potential temperature (water vapor mixing ratio) profile in which the value is 298.15 K/333.15 K (3.00 g kg^−1^/0.67 g kg^−1^) at the surface/model top height is considered for all the experiments. The initial background wind speed is set to 0 m s^−1^ in all vertical layers, and the Coriolis parameter is set to 0 s^−1^. The initial soil moisture content is set to 0.3 m^3^ m^−3^ in all soil layers. The initial passive tracer (CO) concentration is set to 400 ppb at the first model level in the urban area. A diurnally varying passive tracer emission from the surface is considered in the urban area. Its diurnal variation is based on that of the traffic volume in Seoul in 2023 (https://topis.seoul.go.kr), and the daily mean emission rate is 3 × 10^−7^ g m^−2^ s^−1^ (Supplementary Fig. S1). For each experiment, the ensemble simulations are performed following Tabassum et al.^37^: 10 ensemble members are considered, and random potential temperature perturbations within [− 0.1 K, 0.1 K] are applied to the three lowest model levels. The model integration period is from 0000 LST 21 June to 0000 LST 23 June (a summer period). For analysis, the ensemble-mean results for the 24-h period starting from 0000 LST 22 June are used. The UHI intensity is defined as the 2-m temperature averaged over all urban grids minus the 2-m temperature averaged over all rural grids. Referring to Tabassum et al.^37^, the UBC intensity is defined as the maximum horizontal wind speed in the lower half of the PBL. The main analyses are conducted for the daytime which is defined as the period from 1200 to 1700 LST.

## Results and discussions

### Changes in the UHI, UBC, and urban tracer concentration with roof albedo

**Fig. 1:**
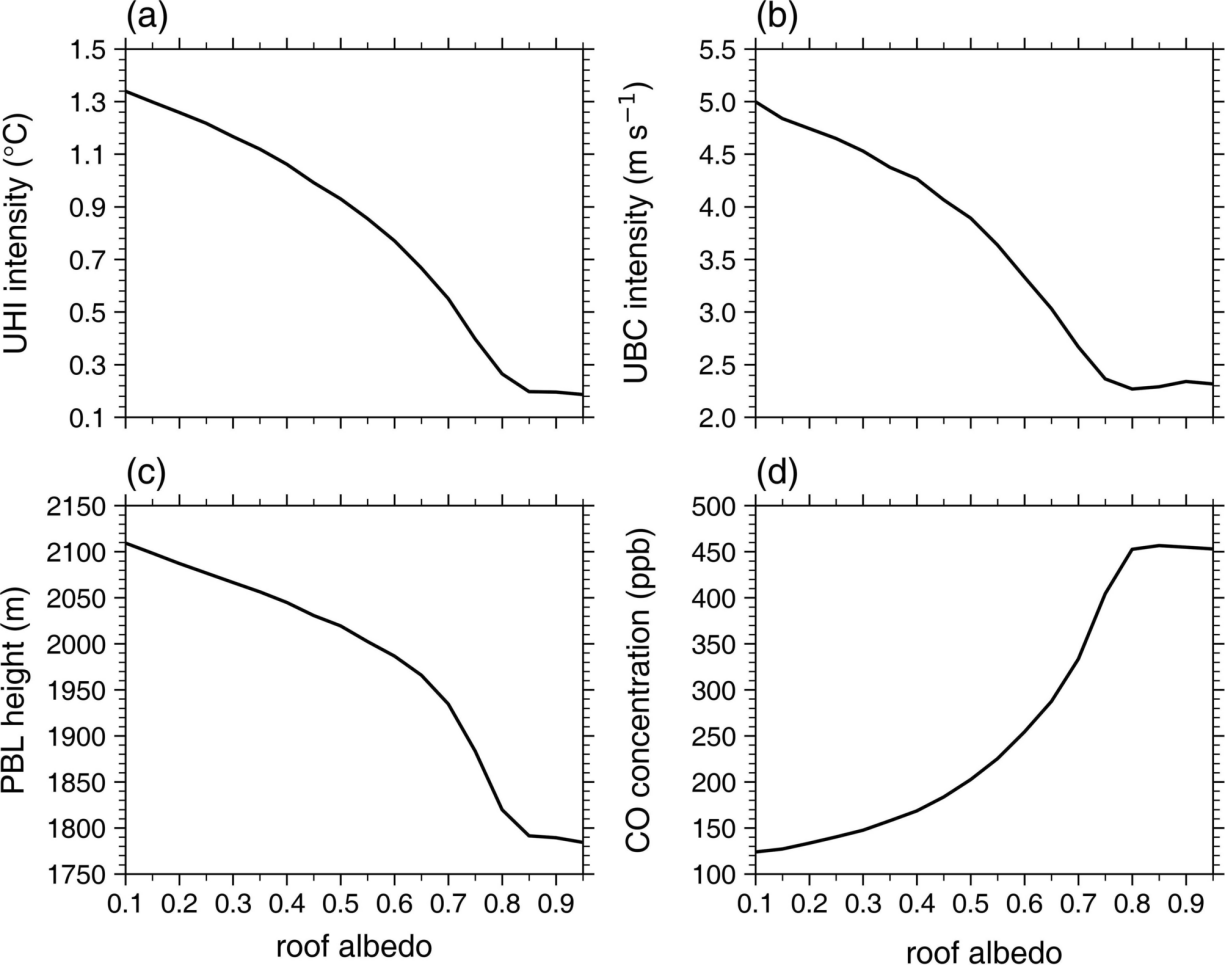
Daytime mean (**a**) urban heat island intensity, (**b**) urban breeze circulation intensity, (**c**) urban planetary boundary layer height, and (**d**) urban passive tracer (carbon monoxide) concentration at the first model level as a function of roof albedo.

Figure 1 shows the daytime mean UHI intensity, UBC intensity, urban PBL height, and urban passive tracer concentration at the first model level as a function of roof albedo (*α*_r_). As can be expected, the daytime mean UHI intensity monotonically decreases with increasing *α*_r_. As *α*_r_ increases from 0.20 to 0.65, the daytime mean UHI intensity decreases from 1.26 °C to 0.67 °C (by 47%). This UHI mitigation effect is comparable to those reported in previous studies^18–21^. It is notable that the rate of decrease in the UHI intensity overall increases with increasing *α*_r_ for 0.10 ≤ *α*_r_ < 0.80. For *α*_r_ ≥ 0.80, the daytime mean UHI intensity very slightly changes with *α*_r_. Thus, in the present idealized simulations, the daytime UHI intensity nonlinearly changes with *α*_r_: The effect of increasing* α*_r_ on the UHI mitigation is enhanced as *α*_r_ increases and becomes very weak when *α*_r_ is very high (*α*_r_ ≥ 0.80). The nonlinear change in the UHI intensity with *α*_r_ is closely associated with those in the daytime mean urban 2-m temperature (Supplementary Fig. S2). The daytime mean rural 2-m temperature very slightly changes with *α*_r_ (Supplementary Fig. S2).

As the daytime mean UHI intensity decreases with increasing *α*_r_, the daytime mean UBC intensity similarly decreases as *α*_r_ increases (Fig. 1b). The similar response of the UBC to an increase in *α*_r_ as that of the UHI is due to the fact that the UBC is driven by the UHI. As *α*_r_ increases from 0.20 to 0.65, the daytime mean UBC intensity decreases from 4.7 m s^−1^ to 3.0 m s^−1^ (by 36%). As *α*_r_ increases, the daytime mean urban PBL height also monotonically decreases (Fig. 1c). This is attributed to the decrease in urban near-surface air temperature with increasing *α*_r_ (Supplementary Fig. S2) which stabilizes the urban PBL^52^. The decrease in daytime PBL height with increasing *α*_r_ has been shown in previous studies^16,17^. As *α*_r_ increases from 0.20 to 0.65, the daytime mean urban PBL height decreases from 2087 m to 1966 m (by 6%). The daytime mean urban PBL height exhibits a nearly linear decrease with increasing *α*_r_ for 0.10 ≤ *α*_r_ ≤ 0.30, but the rate of decrease in the PBL height overall increases with increasing *α*_r_ for 0.30 < *α*_r_ < 0.80. For *α*_r_ ≥ 0.80, the daytime mean urban PBL height very slightly changes with *α*_r_.

The daytime mean urban near-surface tracer concentration generally exhibits a substantial increase with increasing *α*_r_ (Fig. 1d). Given that the UBC transports relatively clean rural air to the urban area and relatively polluted urban air to the rural area, this is primarily due to the overall decrease in the UBC intensity with increasing *α*_r_ (Fig. 1b). Furthermore, the decrease in the urban PBL height with increasing *α*_r_ (Fig. 1c), which indicates the reduction in the vertical turbulent mixing^53^, is also responsible for the overall increase in the urban tracer concentration with increasing *α*_r_. As *α*_r_ increases from 0.20 to 0.65, the daytime mean urban near-surface tracer concentration considerably increases from 133 ppb to 287 ppb (by 115%). Like the UHI intensity, it is noticeable that the rate of increase in the urban tracer concentration generally increases with increasing *α*_r_ for 0.10 ≤ *α*_r_ < 0.80 and the urban tracer concentration exhibits no significant change with *α*_r_ for *α*_r_ ≥ 0.80. This indicates that in the present simulations, the daytime urban tracer concentration also nonlinearly changes with *α*_r_: The negative effect of increasing* α*_r_ on urban air pollutant dispersion is enhanced as *α*_r_ increases and becomes very weak when *α*_r_ is very high (*α*_r_ ≥ 0.80). This result is reproduced in the experiments with the 1000-m horizontal grid interval which does not belong to the gray-zone resolutions^54^ (Supplementary Fig. S3). Overall, the simulated worsening of urban air quality, indicated by the increase in the tracer concentration, with increasing *α*_r_ is notably strong. In the present simulations, the UBC is the sole local circulation affecting the dispersion of air pollutants. In the presence of prevailing background winds and/or other local circulations, the degree of changes in urban air pollutant concentrations with *α*_r_ could greatly differ^24,28^. Furthermore, for secondary air pollutants (e.g., O_3_, PM_2.5_), changes in various temperature-dependent chemical reactions with *α*_r_ can affect the impact of increasing *α*_r_ on pollutant concentrations in complex ways^27,55^, which deserves further investigations.

**Fig. 2:**
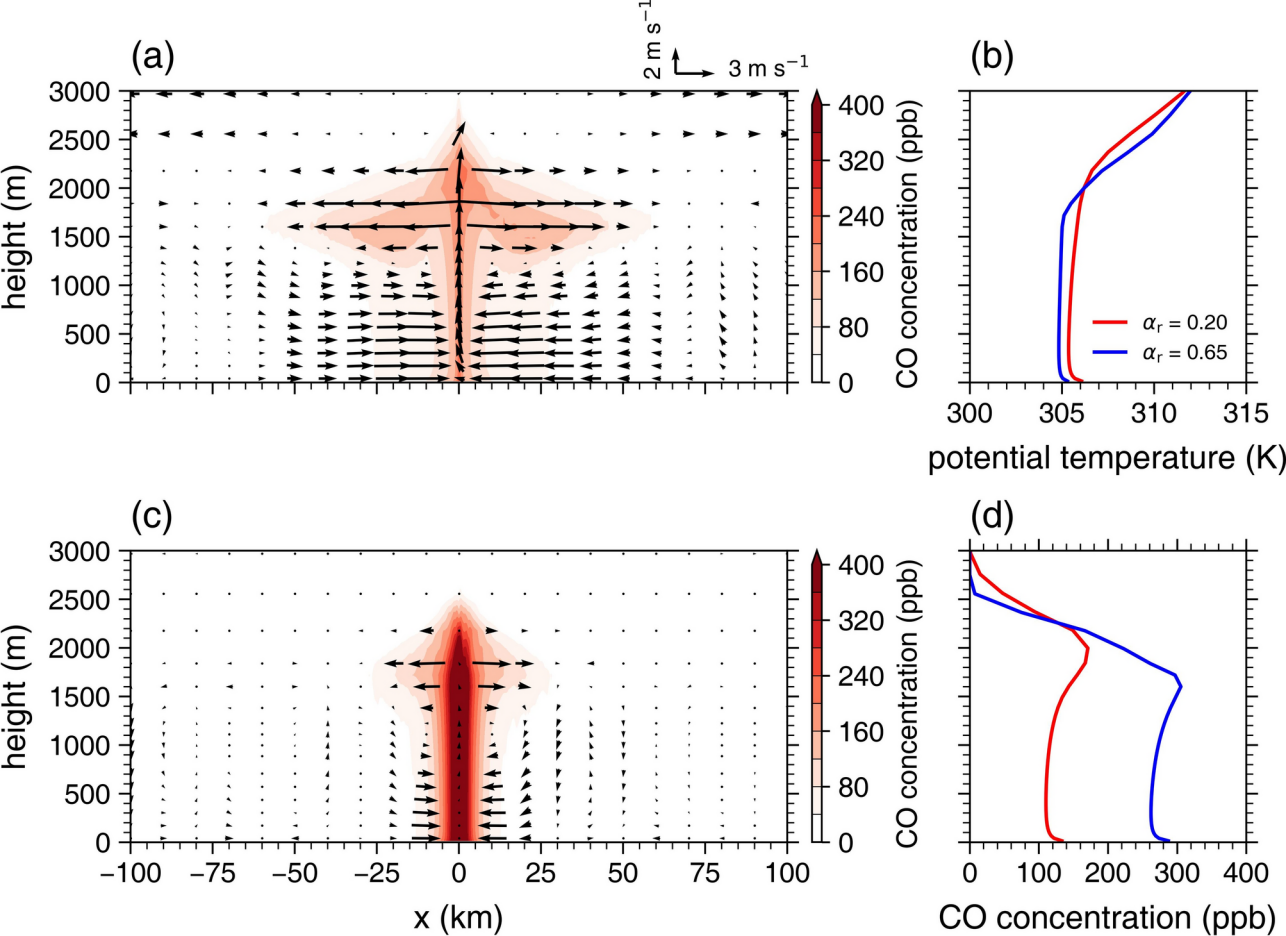
(**a**) Daytime mean field of passive tracer (carbon monoxide) concentration (shade) and wind vectors (arrow) for a roof albedo of 0.20. (**b**) Daytime mean vertical profiles of urban potential temperature for roof albedos of 0.20 (red) and 0.65 (blue). (**c**) Same as (**a**) except for a roof albedo of 0.65. (**d**) Daytime mean vertical profiles of urban passive tracer concentration for roof albedos of 0.20 (red) and 0.65 (blue).

How increasing *α*_r_ affects the structures of the UBC and urban PBL is examined in detail. Figure 2 shows the daytime mean fields of wind vectors and passive tracer concentration and the daytime mean vertical profiles of urban potential temperature and urban tracer concentration for *α*_r_ = 0.20 and 0.65. For *α*_r_ = 0.20, the UBC is clearly seen, with the converging flows toward the urban area in the lower PBL, the ascending flows at the urban center, and the diverging flows from the urban area in the upper PBL (Fig. 2a). The horizontal size of the UBC is 125 km, and the vertical size of the UBC is 3.0 km at the urban center. The maximum vertical velocity at the urban center is 4.1 m s^−1^. Along the well-developed UBC, the tracer is widely dispersed. As *α*_r_ increases to 0.65, the weakening and contraction of the UBC are evident (Fig. 2c). The horizontal size of the UBC decreases to 51 km, and the vertical size of the UBC decreases to 2.6 km at the urban center. Furthermore, the maximum vertical velocity at the urban center considerably decreases to 0.5 m s^−1^. As a result, the dispersion of the tracer is greatly inhibited, the tracer being accumulated within the PBL at and near the urban center.

The increase in* α*_r_ leads to significant changes in urban potential temperature and tracer concentration throughout the PBL (Fig. 2b and d). For *α*_r_ = 0.20, a statically unstable layer in which the rate of change in potential temperature with height is − 0.014 K m^−1^ is found below *z* = 40 m (Fig. 2b). Above *z* ~ 0.1 km, a well-mixed layer is developed up to *z* ~ 2.0 km. The daytime mean potential temperature averaged over the well-mixed layer is 305.5 K. As *α*_r_ increases to 0.65, the static instability below *z* = 40 m (− 0.009 K m^−1^) decreases and the top height of the well-mixed layer decreases to *z* ~ 1.8 km. This inhibits urban air pollutants from being dispersed away from the surface, contributing to the increase in the urban tracer concentration (Fig. 1d). The daytime mean urban potential temperature averaged over the well-mixed layer decreases by 0.6 K, which is comparable to the decrease in the UHI intensity (Fig. 1a). This urban PBL cooling by the increase in *α*_r_ agrees with previous studies^16,17,31^. Meanwhile, as *α*_r_ increases from 0.20 to 0.65, the urban tracer concentration increases throughout the PBL (Fig. 2d). The daytime mean urban tracer concentration averaged over the well-mixed layer increases from 118 ppb to 270 ppb.

### Changes in roof surface temperature and roof surface energy fluxes with roof albedo and their associations with the nonlinear change in UHI intensity

**Fig. 3:**
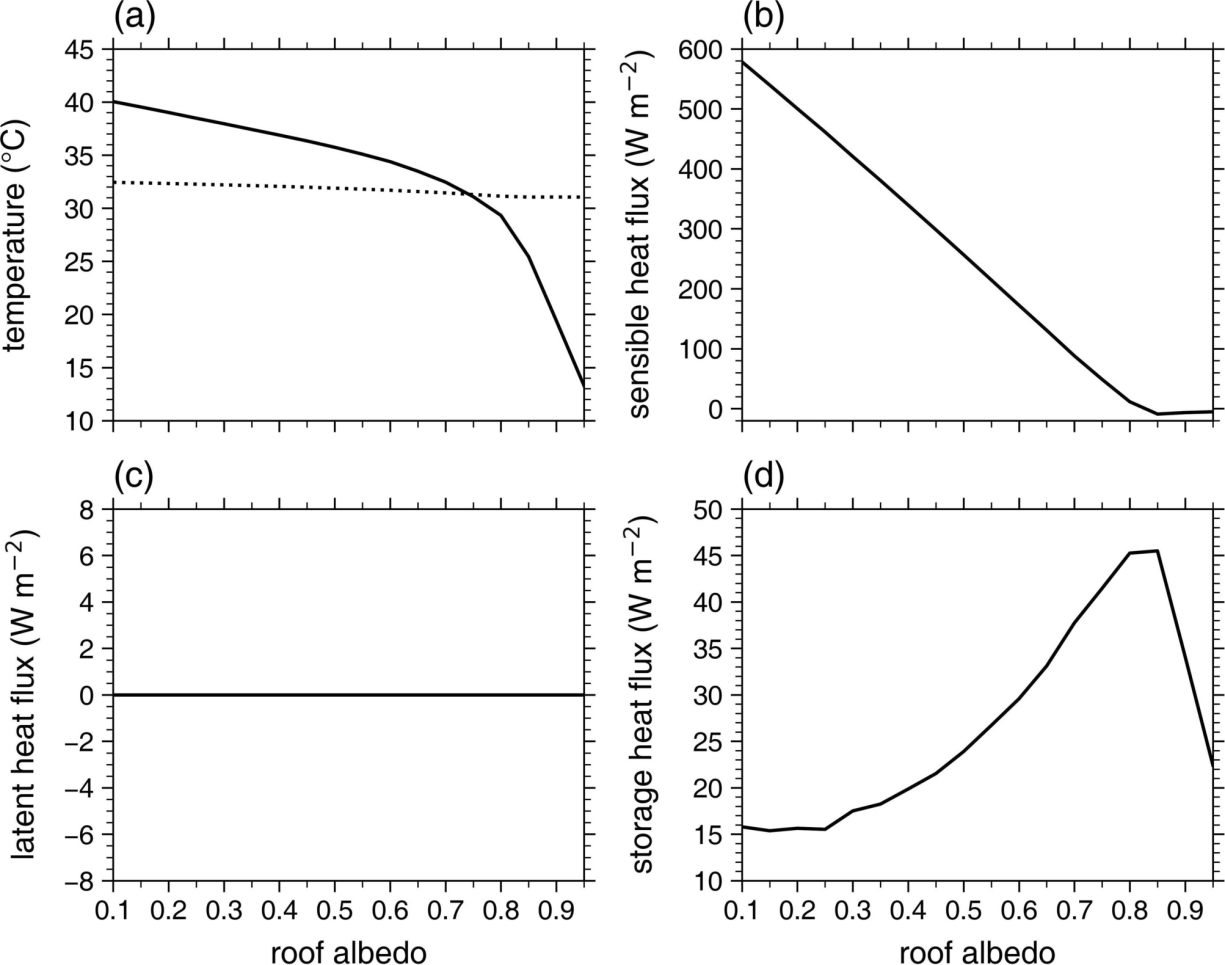
Daytime mean (**a**) roof surface temperature, (**b**) roof sensible heat flux, (**c**) roof latent heat flux, and (**d**) roof storage heat flux as a function of roof albedo. The dotted line in (**a**) indicates the daytime mean urban 2-m temperature as a function of roof albedo.

Given changes in *α*_r_ directly alter the roof surface energy balance and thus roof surface temperature, changes in UHI intensity with *α*_r_ are primarily associated with those in roof surface temperature and roof surface energy fluxes. In this subsection, to elucidate the nonlinear change in the daytime UHI intensity with *α*_r_ (Fig. 1a), how the roof surface temperature and roof surface energy fluxes change with *α*_r_ is examined. Figure 3 shows the daytime mean roof surface temperature, roof sensible heat flux, roof latent heat flux, and roof storage heat flux as a function of *α*_r_. The daytime mean roof surface temperature monotonically decreases with increasing *α*_r_ (Fig. 3a). As *α*_r_ increases from 0.20 to 0.65, the daytime mean roof surface temperature decreases from 39.0 °C to 33.5 °C. For 0.10 ≤ *α*_r_ < 0.80, the rate of decrease in the roof surface temperature overall increases with increasing *α*_r_, in line with that in the urban 2-m temperature (Supplementary Fig. S2). This indicates that the nonlinear decrease in the UHI intensity with increasing *α*_r_ for 0.10 ≤ *α*_r_ < 0.80 is associated with that in the roof surface temperature. The rate of decrease in the roof surface temperature also increases with increasing *α*_r_ for *α*_r_ ≥ 0.80, but it is well seen that the daytime mean roof surface temperature is notably lower than the daytime mean urban 2-m temperature (Fig. 3a). The daytime mean roof sensible heat flux almost linearly decreases with increasing *α*_r_ for 0.10 ≤ *α*_r_ < 0.80 (Fig. 3b). As *α*_r_ increases from 0.20 to 0.65, the daytime mean roof sensible heat flux decreases from 500 W m^−2^ to 131 W m^−2^. For *α*_r_ ≥ 0.80, the daytime mean roof sensible heat flux is very small and very slightly changes with *α*_r_. Particularly, the daytime mean roof sensible heat flux is negative for *α*_r_ ≥ 0.85, indicating the sensible heat transfer from the urban near-surface air to the roof surface. Thus, as the roof surface temperature becomes notably lower than the urban near-surface air temperature in the daytime for *α*_r_ ≥ 0.80 (Fig. 3a), the upward sensible heat flux from the roof hardly appears and does not significantly change with *α*_r_ (Fig. 3b). Furthermore, due to the notably lower roof surface temperature than the urban near-surface air temperature, the net radiative heat transfer from the roof surface to the urban near-surface air can also be greatly inhibited. These may greatly weaken the decrease in the UHI intensity with increasing *α*_r_ for *α*_r_ ≥ 0.80 (Fig. 1a). Meanwhile, the daytime mean roof latent heat flux is 0 W m^−2^ for all *α*_r_ (Fig. 3c). This is due to the lack of roof vegetation. Note that the urban latent heat flux arises from the natural area fraction in the urban grid.

The daytime mean roof storage heat flux exhibits distinct changes from the roof sensible and latent heat fluxes as *α*_r_ increases (Fig. 3d). The daytime mean roof storage heat flux is positive for all *α*_r_, indicating that the heat is conducted from the roof surface to the roof subsurface (heat storing). Interestingly, for 0.10 ≤ *α*_r_ < 0.80, the daytime mean roof storage heat flux overall increases with increasing *α*_r_. As *α*_r_ increases from 0.20 to 0.65, the daytime mean roof storage heat flux increases from 16 W m^−2^ to 33 W m^−2^. Furthermore, the rate of increase in the roof storage heat flux overall increases with increasing *α*_r_. Since the heat storing from the roof surface into the roof subsurface inhibits the roof surface heating by solar radiation, the overall increasing rate of increase in the roof storage heat flux with increasing *α*_r_ for 0.10 ≤ *α*_r_ < 0.80 is responsible for that of decrease in the roof surface temperature (Fig. 3a). On the other hand, for *α*_r_ ≥ 0.80, the daytime mean roof storage heat flux overall linearly decreases with increasing *α*_r_ (Fig. 3d).

**Fig. 4:**
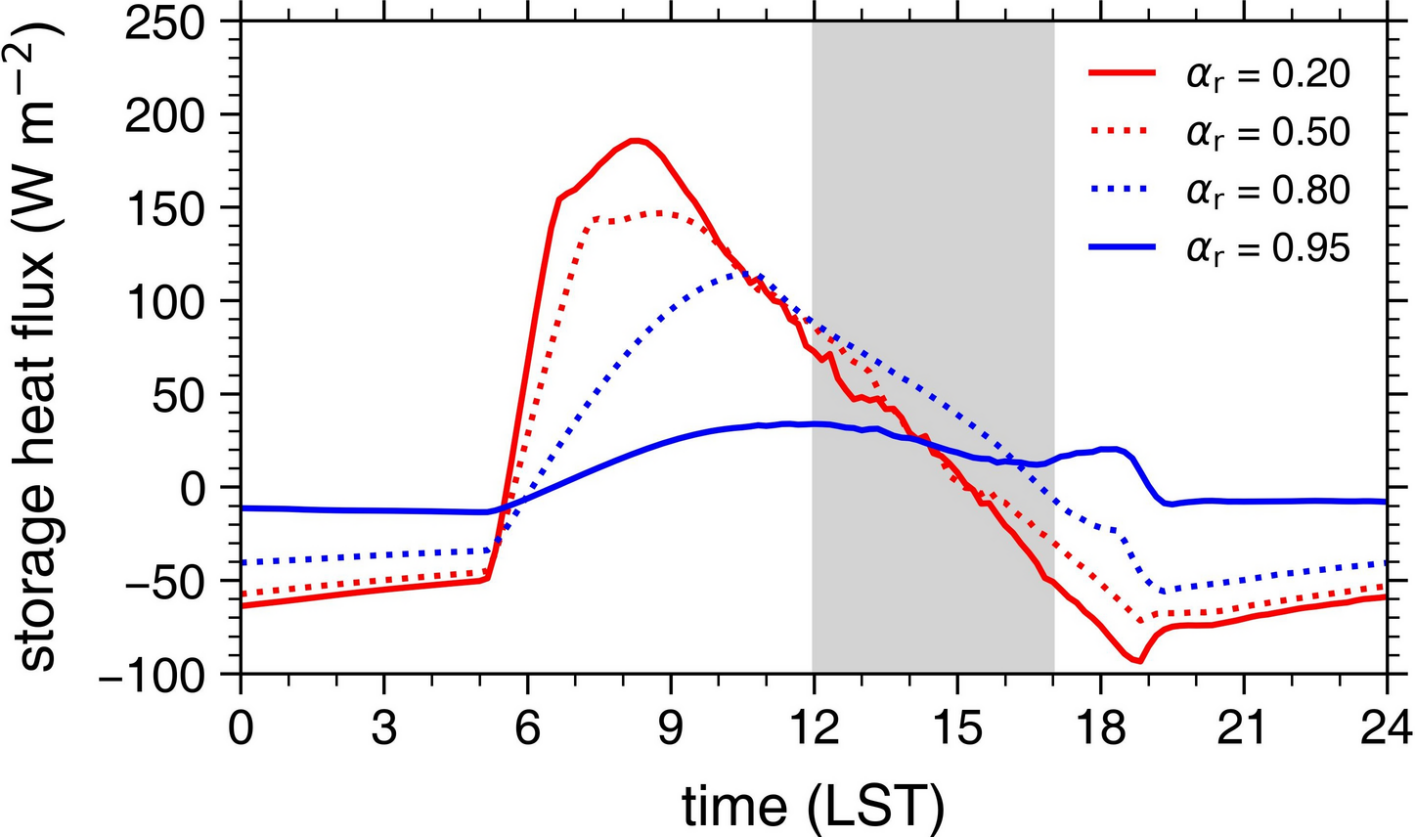
Diurnal variations of roof storage heat flux for roof albedos of 0.20 (red solid line), 0.50 (red dotted line), 0.80 (blue dotted line), and 0.95 (blue solid line). The shaded area indicates the daytime defined as the period between 1200 and 1700 LST.

Changes in roof storage heat flux with *α*_r_ are further examined in Fig. 4. Figure 4 shows the diurnal variations of roof storage heat flux for *α*_r_ = 0.20, 0.50, 0.80, and 0.95. For *α*_r_ = 0.20, the maximum roof storage heat flux is 186 W m^−2^ and appears at 0820 LST (Fig. 4). This shows that the heat storing into the roof subsurface mainly occurs in the morning. It is well known that the net radiation at the urban surface is primarily transferred into storage heat flux in the morning and into sensible heat flux in the daytime^56^. As *α*_r_ increases from 0.20 to 0.50, the mean roof storage heat flux in the morning (from 0600 to 1100 LST) decreases by 27 W m^−2^ while the daytime mean roof storage heat flux increases by 8 W m^−2^. This indicates that the period when the heat storing mainly occurs is delayed as* α*_r_ increases. As *α*_r_ further increases from 0.50 to 0.80, the mean roof storage heat flux in the morning further decreases by 49 W m^−2^ while the daytime mean roof storage heat flux further increases by 21 W m^−2^. The rate of decrease in the mean roof storage heat flux in the morning overall increases with increasing *α*_r_ for 0.10 ≤ *α*_r_ < 0.80 (Supplementary Fig. S4), similar to the rate of increase in the daytime mean roof storage heat flux (Fig. 3d). Thus, the above results suggest that for 0.10 ≤ *α*_r_ < 0.80, a low to moderately high *α*_r_ range, the increase in *α*_r_ may cause the solar radiation absorbed at the roof surface in the daytime to be more transferred into storage heat flux due to the lack of heat storing into the roof subsurface in the morning. On the other hand, as *α*_r_ increases from 0.80 to 0.95, the roof storage heat flux decreases in both morning and daytime and becomes very small (Fig. 4). This suggests that for *α*_r_ ≥ 0.80, a very high *α*_r_ range, the increase in *α*_r_ no more increases roof storage heat flux in the daytime because of too small amount of solar radiation absorbed at the roof surface in the daytime to be transferred into storage heat flux. For *α*_r_ ≥ 0.80, the roof storage heat flux overall linearly decreases with increasing *α*_r_ in both morning and daytime (Fig. 3d and Supplementary Fig. S4). It should be noted that the value of *α*_r_ from which the change in the roof storage heat flux with *α*_r_ differs could vary with urban thermal and morphological characteristics and background meteorological conditions. To understand the physical mechanisms behind changes in roof surface energy fluxes with *α*_r_, further examinations for various urban configurations and meteorological conditions are needed.

**Fig. 5:**
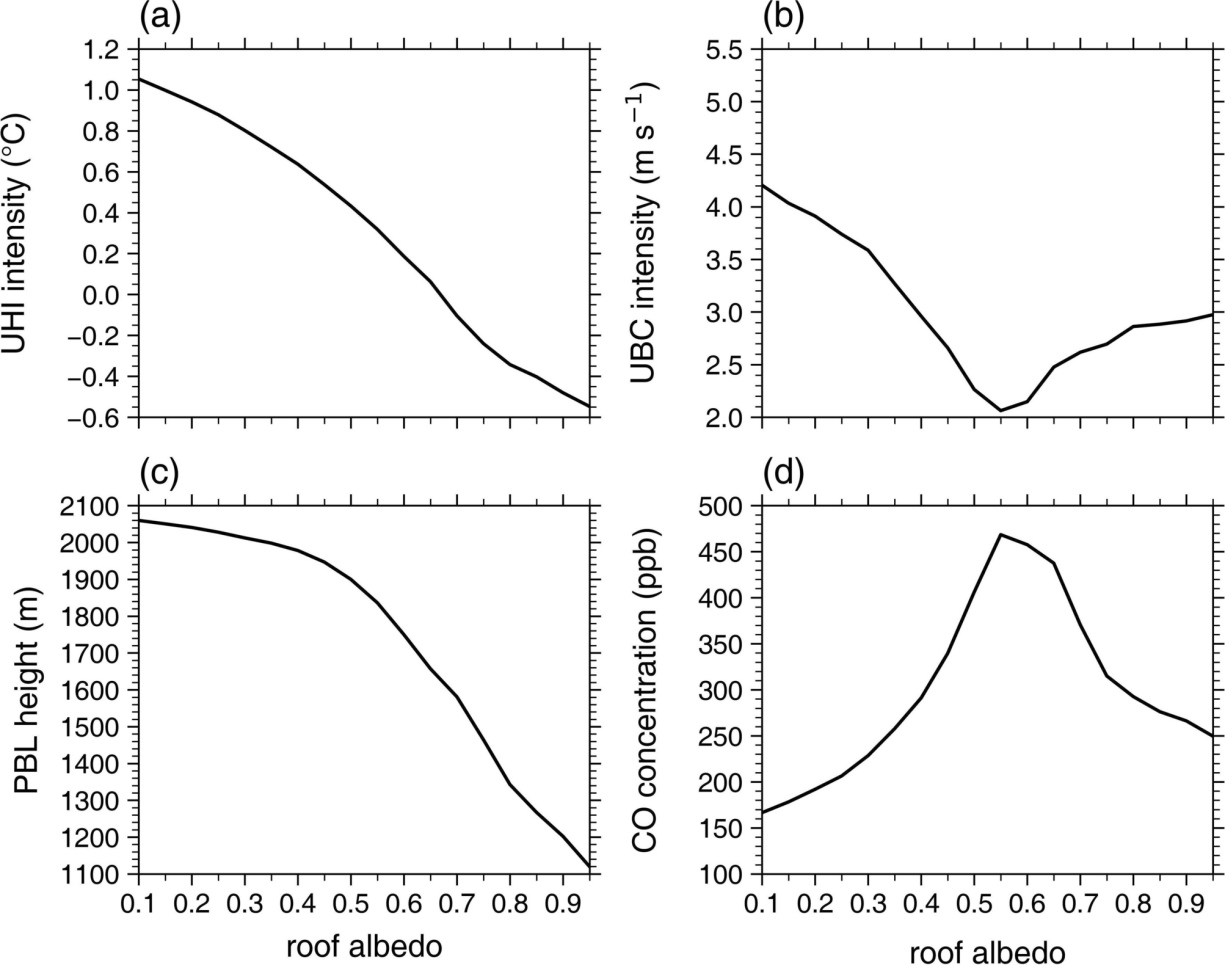
Same as Fig. 1 except for the experiments with the SLUCM.

Finally, to examine how changes in the UHI intensity, UBC intensity, and urban tracer concentration with *α*_r_ differ depending on UCMs, additional experiments are performed by using a single-layer UCM (SLUCM) developed by Kusaka et al.^57^ instead of the SNUUCM. Figure 5 is the same as Fig. 1 except for the experiments with the SLUCM. Compared to the experiments with the SNUUCM, the daytime mean UHI intensity in the experiments with the SLUCM is weaker for all* α*_r_ and more decreases with increasing *α*_r_ (Fig. 5a). The daytime mean UHI intensity becomes negative for *α*_r_ ≥ 0.70, indicating the urban cool island. Due to this, the reversed UBC appears for *α*_r_ ≥ 0.70 and its intensity increases with increasing *α*_r_ (Fig. 5b). As *α*_r_ increases, the daytime mean urban PBL height in the experiments with the SLUCM also more decreases than that in the experiments with the SNUUCM (Fig. 5c). The daytime mean urban near-surface tracer concentration increases as the UBC intensity decreases (Fig. 5d), showing a maximum concentration when the UBC intensity is weakest (*α*_r_ = 0.55). Interestingly, the nonlinearity of change in the UHI intensity with *α*_r_ is relatively weak in the experiments with the SLUCM (Fig. 5a). Furthermore, the energy partitioning and changes in roof surface energy fluxes with *α*_r_ in the experiments with the SLUCM are quite different from those in the experiments with the SNUUCM. In comparison with the experiments with the SNUUCM, the daytime roof sensible heat flux is generally smaller in the experiments with the SLUCM, while the daytime roof storage heat flux is generally larger (Supplementary Fig. S5). It is also notable that for 0.10 ≤ *α*_r_ < 0.80, the daytime roof storage heat flux in the experiments with the SLUCM does not increase with increasing *α*_r_ and the rate of decrease in the daytime roof sensible heat flux overall slightly decreases with increasing *α*_r_ (Supplementary Fig. S5). These differing energy partitioning and changes in roof surface energy fluxes depending on the UCMs make changes in UHI intensity with *α*_r_ and their associated physical processes vary. These results imply the necessity of evaluating how the UHI intensity changes with *α*_r_ with various UCMs to better understand the relationship between the UHI and* α*_r_.

## Conclusions

**Fig. 6:**
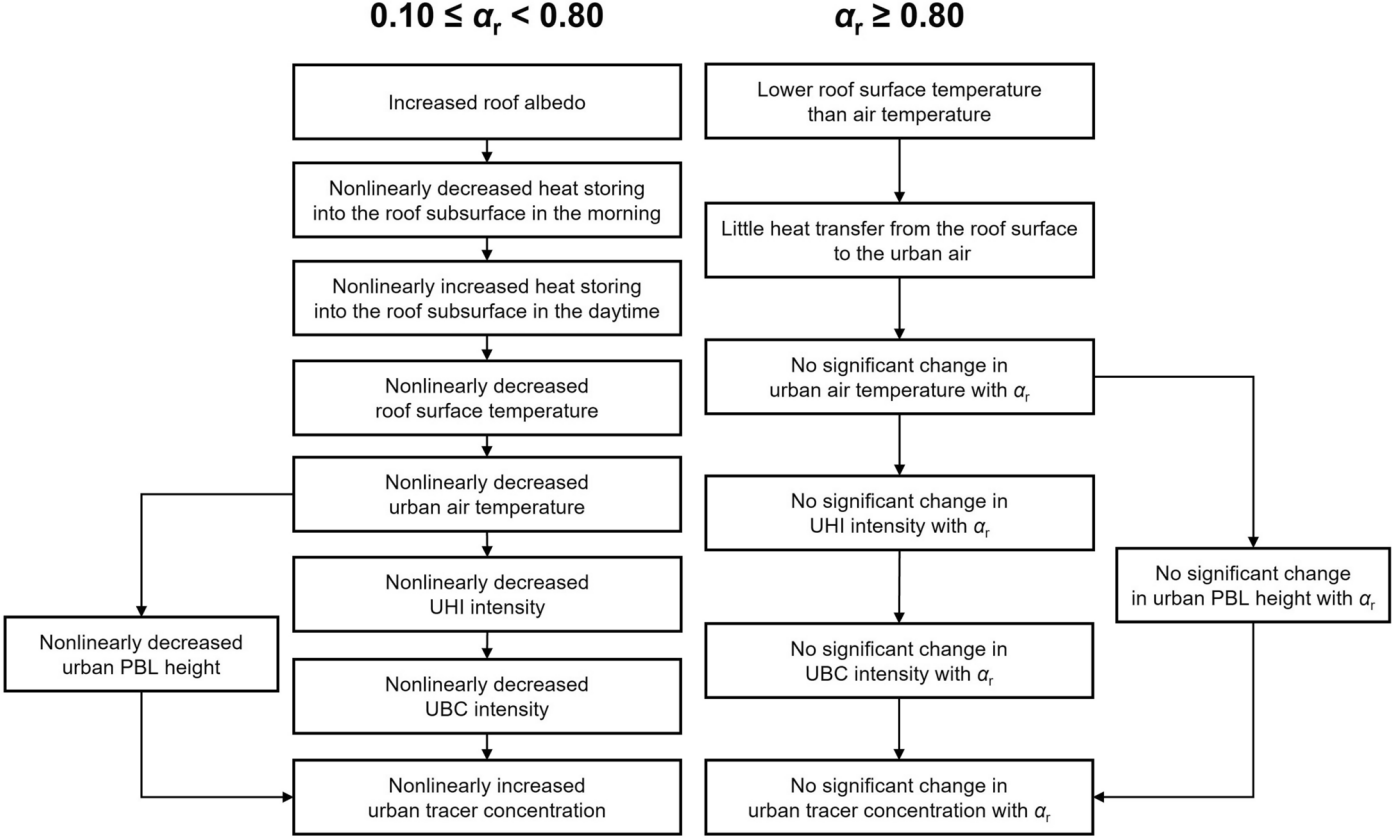
Schematic diagram of the impacts of increasing roof albedo on the urban heat island, urban breeze circulation, and urban tracer concentration found in the idealized ensemble simulations.

In this study, changes in the UHI, UBC, and urban air pollutant dispersion with *α*_r_ are systematically examined through idealized ensemble simulations with the WRF model coupled with the SNUUCM. As *α*_r_ increases, the daytime mean UHI intensity, UBC intensity, and urban PBL height overall decrease and the daytime mean urban near-surface passive tracer concentration generally exhibits a substantial increase with increasing *α*_r_. The daytime mean UHI intensity, UBC intensity, and urban tracer concentration nonlinearly change with *α*_r_. For 0.10 ≤ *α*_r_ < 0.80, the effects of increasing *α*_r_ on the UHI, UBC and urban air pollutant dispersion are overall enhanced as* α*_r_ increases. For *α*_r_ ≥ 0.80, the roof surface temperature is notably lower than the urban near-surface air temperature in the daytime, the effects of increasing *α*_r_ on the UHI, UBC and urban air pollutant dispersion being very weak. The nonlinear changes in the UHI intensity, UBC intensity, and urban tracer concentration with *α*_r_ are associated with the changes in the roof surface temperature and roof surface energy fluxes with *α*_r_, as presented in Fig. 6.

In this study, it is found that changes in the UHI intensity, UBC intensity, and urban tracer concentration with *α*_r_ for 0.10 ≤ *α*_r_ < 0.80 are considerably different from those for *α*_r_ ≥ 0.80. However, the value of *α*_r_ from which these changes differ may vary depending on urban thermal and morphological characteristics and background meteorological conditions. To advance the understanding of changes in UHI intensity with *α*_r_ and their associated physical processes, further investigations with simulations under different urban characteristics, seasons, and latitudes are required. Furthermore, it should be noted that changes in UHI intensity with *α*_r_ and their associated physical processes also vary depending on UCMs. This is attributed to that each UCM simulates urban turbulent and storage heat fluxes differently for given incoming solar radiation and meteorological conditions. Given this aspect, the use of various UCMs could help to better understand the relationship between the UHI and *α*_r_.

## Supplementary Information

Supplementary Figures.

## Data Availability

The datasets generated during and/or analyzed during the current study are available from the corresponding author on reasonable request.
