# Supplementary material for: Nonlinear changes in urban heat island intensity, urban breeze intensity, and urban air pollutant concentration with roof albedo
Source: Sci Rep. 2024 Oct 22;14:24911. doi: 10.1038/s41598-024-76935-4 (PMC11496832; doi:10.1038/s41598-024-76935-4)
Supplement: Supplementary file 1 — Supplementary Figures. [file 41598_2024_76935_MOESM1_ESM.docx]

**Supplementary information for**

**Nonlinear changes in urban heat island intensity, urban breeze intensity, and urban air pollutant concentration with roof albedo**

Kyeongjoo Park^1^ & Jong-Jin Baik^1*^

^1^School of Earth and Environmental Sciences, Seoul National University, Seoul 08826, South Korea

^*^Corresponding author:

Prof. Jong-Jin Baik

Email: jjbaik@snu.ac.kr

**
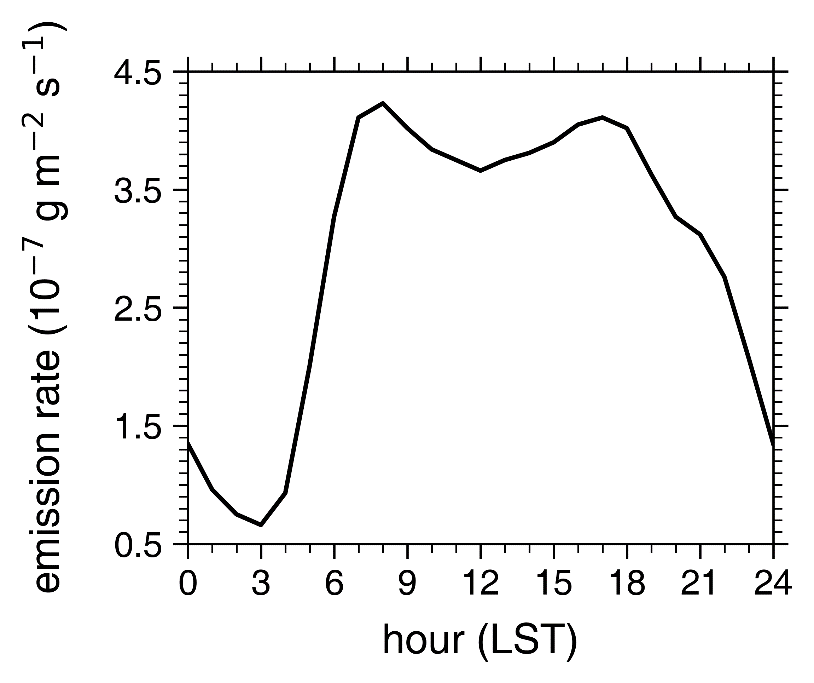
**

**Figure S1.** Diurnal variation of the emission rate of the passive tracer (carbon monoxide).

**
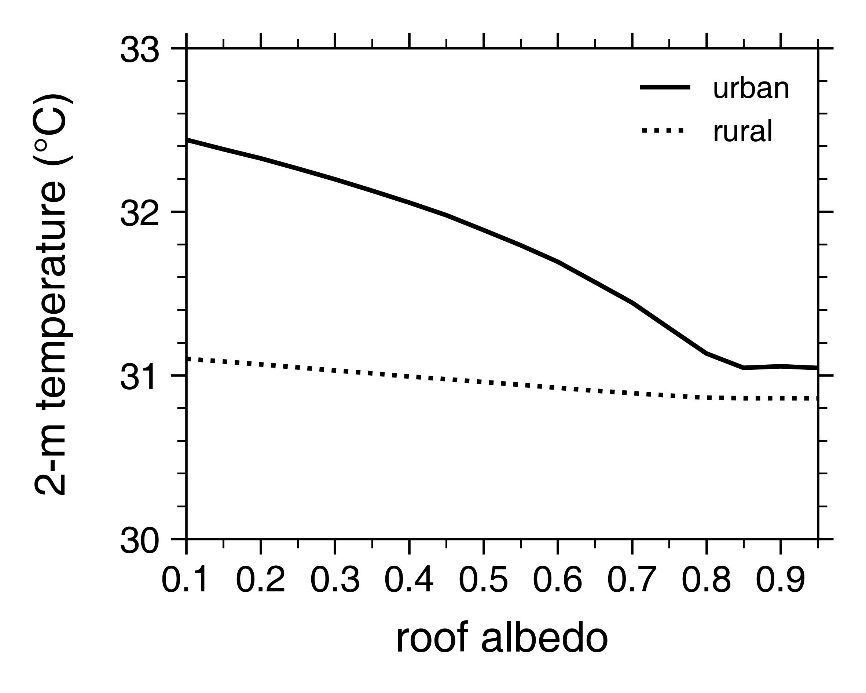
**

**Figure S2.** Daytime mean urban (solid) and rural (dotted) 2-m temperatures as a function of roof albedo.

**
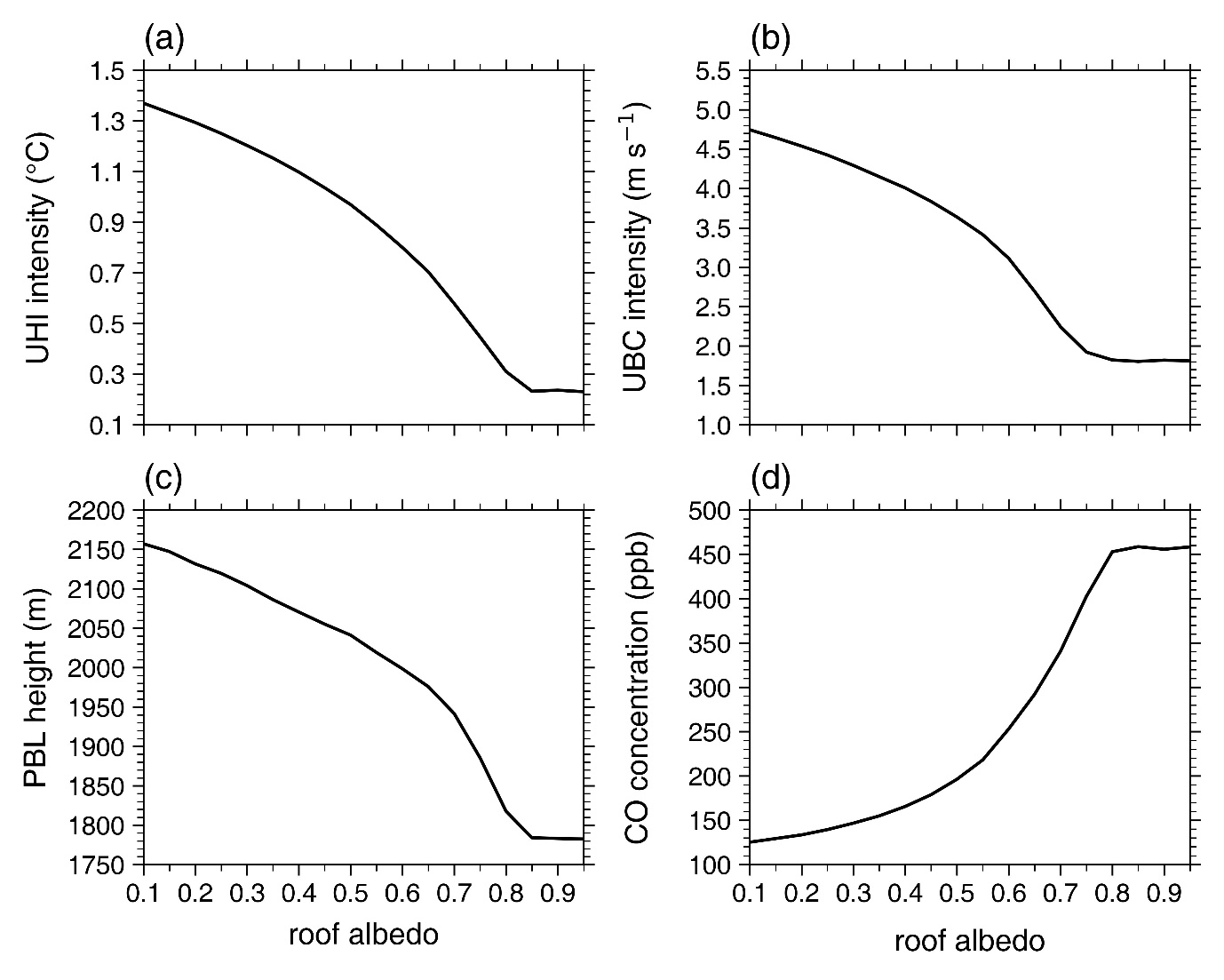
**

**Figure S3.** Same as Fig. 1 except for the experiments with the 1000-m horizontal grid interval.


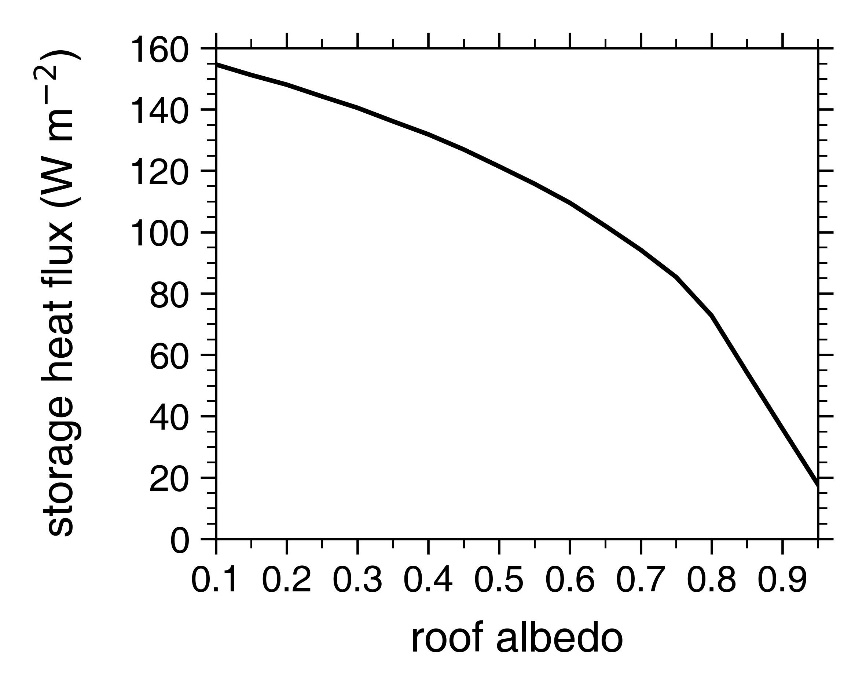


**Figure S4.** Same as Fig. 3d except for the mean roof storage heat flux from 0600 LST to 1100 LST.


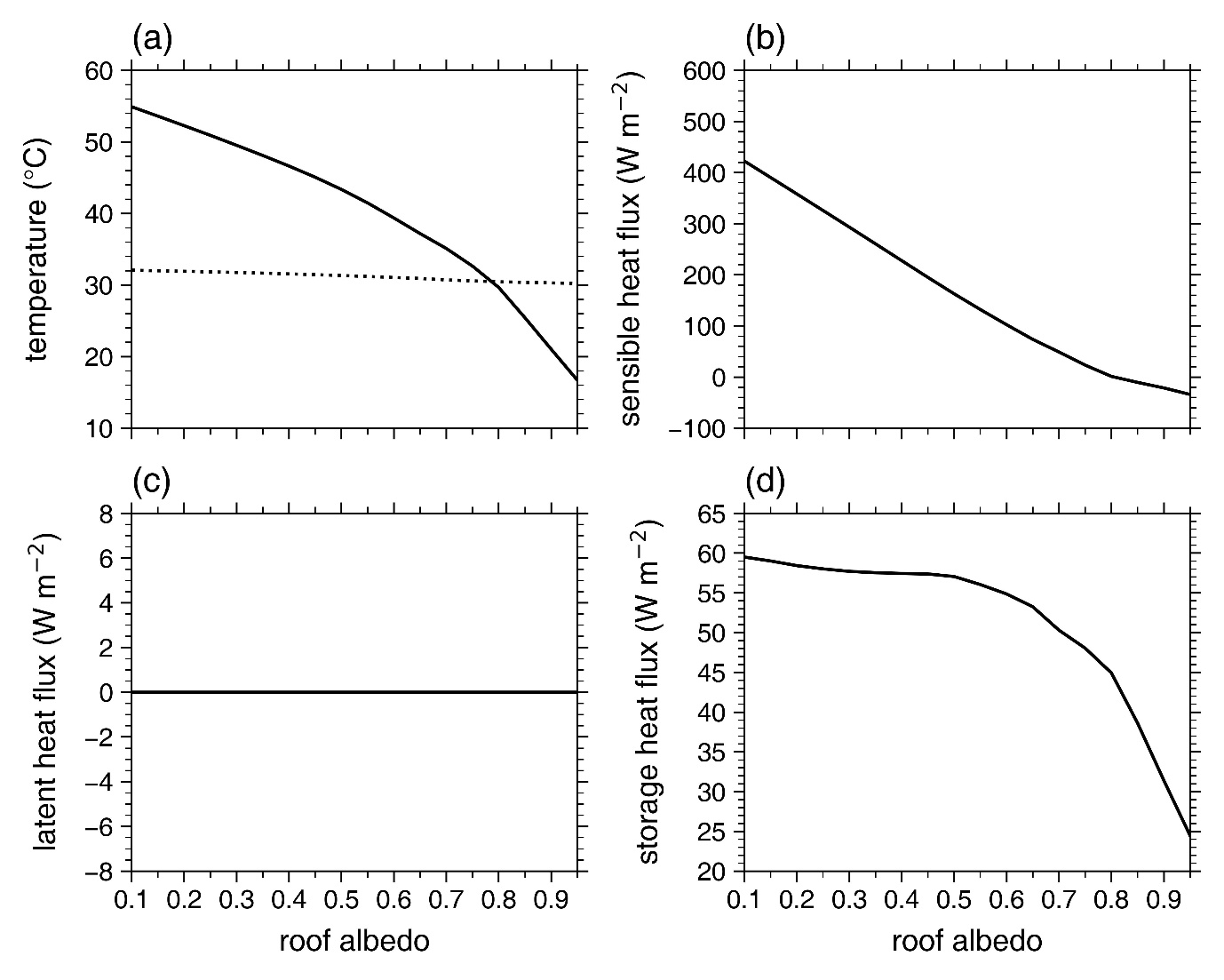


**Figure S5.** Same as Fig. 3 except for the experiments with the SLUCM.
